# An Overview of the Stability and Delivery Challenges of Commercial Nucleic Acid Therapeutics

**DOI:** 10.3390/pharmaceutics15041158

**Published:** 2023-04-06

**Authors:** Rahul G. Ingle, Wei-Jie Fang

**Affiliations:** 1 Institute of Drug Metabolism and Pharmaceutical Analysis, College of Pharmaceutical Sciences, Zhejiang University, Hangzhou 310027, China; 2 Dr. Rajendra Gode College of Pharmacy, Amravati 444602, India

**Keywords:** drug delivery, excipient, formulation, mRNA vaccine, nucleic acid therapeutics, stability

## Abstract

Nucleic acid (NA)-based biopharmaceuticals have emerged as promising therapeutic modalities. NA therapeutics are a diverse class of RNA and DNA and include antisense oligonucleotides, siRNA, miRNA, mRNA, small activating RNA, and gene therapies. Meanwhile, NA therapeutics have posed significant stability and delivery challenges and are expensive. This article discusses the challenges and opportunities for achieving stable formulations of NAs with novel drug delivery systems (DDSs). Here we review the current progress in the stability issues and the significance of novel DDSs associated with NA-based biopharmaceuticals, as well as mRNA vaccines. We also highlight the European Medicines Agency (EMA) and US Food and Drug Administration (FDA)-approved NA-based therapeutics with their formulation profiles. NA therapeutics could impact future markets if the remaining challenges and requirements are addressed. Regardless of the limited information available for NA therapeutics, reviewing and collating the relevant facts and figures generates a precious resource for formulation experts familiar with the NA therapeutics’ stability profile, their delivery challenges, and regulatory acceptance.

## 1. Introduction

Biopharmaceuticals are at the supreme level of the pharmaceutical market due to their high efficacy, high specificity, and low toxicity profiles [1]. Recently, nucleic acid (NA) therapeutics have emerged as promising candidates for several severe diseases and disorders. NAs are present in all living organisms, including humans, animals, and plants [2]. NAs are naturally occurring chemical compounds; certain small NAs are also synthesized in the laboratory. NAs can be broken down into sugars, phosphoric acid, and a mixture of organic bases (e.g., purines and pyrimidines). NAs have been developed as therapeutic agents and carefully characterized to provide the intended quality, efficacy, and safety profile. NAs are complex and delicate molecules that require sophisticated processes with clever handling during manufacturing, which makes these drugs more expensive. The stability of NAs during manufacturing, handling, shipping, and long-term storage is a major subject of discussion. Excipients play a key role in designing NA therapeutics by improving the manufacturability, stability, quality, and safe delivery of the products [3].

Due to their complex nature, NAs require special attention as active pharmaceutical ingredients (APIs). The alteration in NA quality as a result of physicochemical degradation makes their formulation development challenging. Therefore, several aspects must be considered, including active drug concentration, excipients, delivery routes, and novel drug delivery systems (DDSs). The use of excipients at optimized concentrations aims to maintain the stability of NA therapeutics [4]. However, a key obstacle for the formulation expert is to formulate stable NA therapeutics with the narrow range of excipients usually employed in parenteral settings. Therefore, the launch of novel and ideal excipients to maintain the integrity of significant scientific contribution. Specifically, a critical manufacturing hurdle is precision in the reproducibility of chemical synthesis, the assurance of reproducibility, and the integrity of subsequent batches of the NA therapeutics. For example, the synthesis of thiophosphate derivatives of oligonucleotides results in a mixture of 2^n^ diastereomers, in which each diastereomer might interact in a slightly different manner. Here, chirality impacts the physical and biological properties of NAs, such as the binding affinity, nuclease stability, etc. [5]. In addition, NA therapeutics are still related with the dilemma of complex drug delivery. This is a fundamental setback preventing the widespread implementation of NA therapeutics. Naked NAs are quickly degraded into physiological fluids and do not accumulate in target tissues [6,7,8]. Despite these issues, the current NA dosage forms and novel DDSs have enabled the successful launch of NA therapeutics globally. The application of novel DDSs not only improves the long-term storage stability of NAs but also preserves their in vivo efficacy. Therefore, it could be assumed that it is important to conduct an up-to-date survey of the excipients in approved NA therapeutics with novel DDSs. It could serve the biopharmaceutical industry by minimizing the time spent on pre-formulation studies and speeding up the development of stable NA formulations.

Between 2004 and 2021, there have been 23 NA therapeutics approved via the United States Food and Drug Administration (FDA) and/or the European Medicines Agency (EMA) approaches. Among these, fomivirsen (Vitravene) was removed from the European and US markets in 2002 and 2006, respectively, owing to the demand having been undermined. In addition, Macugen (pegaptanib sodium injection), Glybera (alipogene tiparvovec), and Kynamro (mipomersen sodium) were withdrawn from the market in 2019, 2017, and 2022, respectively [9]. Therapeutic NAs formulated in liquid, suspension, and freeze-dried forms, as well as vector-like liposomes/lipids and nanoparticles (NPs), are divided into their functional classes. Antisense oligonucleotides are a major division of approved NA therapeutics. On the other hand, gene therapy has revealed exciting treatment opportunities for numerous severe and rare diseases that have not been cured thus far, although the safety of gene therapy is a major concern. Continuous monitoring is needed to overcome the challenges posed by these new drugs and to increase their contribution as novel therapeutic modalities in the biopharmaceutical industry [10].

To date, one of the major unresolved issues for approved NAs is the high cost of these drugs. For example, nusinersen costs USD $750,000 for the first year and USD $375,000 in subsequent years. Likewise, eteplirsen costs USD $300,000 annually. The expense for high-efficacy, life-saving drugs, such as nusinersen, is likely to be acceptable. In contrast, eteplirsen has a narrow efficacy; therefore, justifying the cost of eteplirsen would be difficult [11,12].

## 2. Lasting Challenges and Considerations of NA Therapeutics

### 2.1. NA Therapeutics Stability

**Figure 1:**
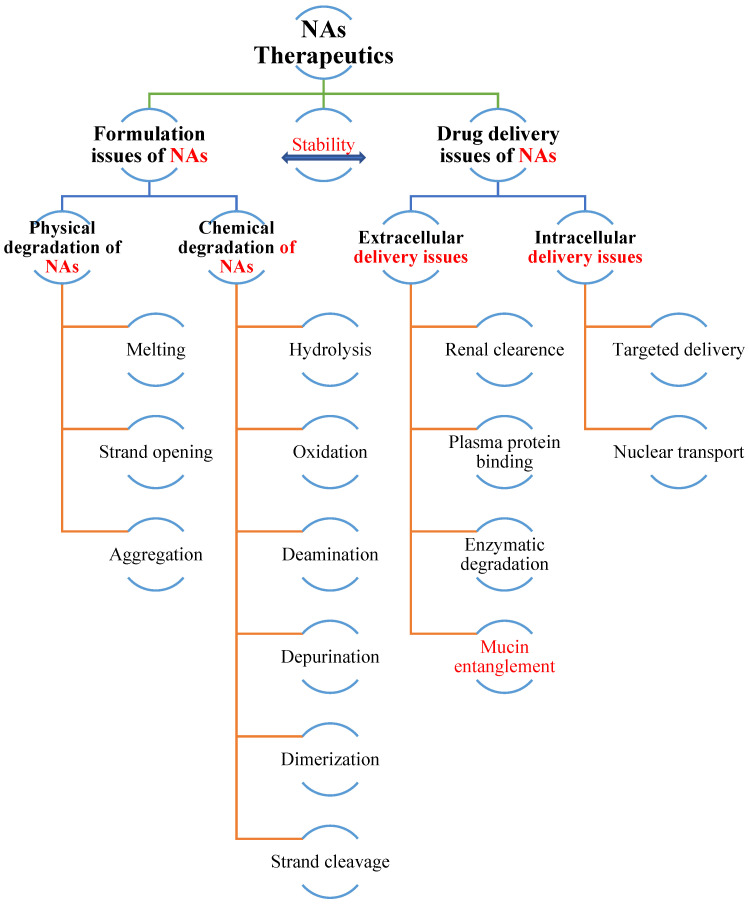
Challenges in the formulation and delivery of NA therapeutics [34].

NAs could have unique stability issues, similar to protein drugs, due to their complex and fragile nature (Figure 1). Naked or unmodified NAs have extremely short half-lives in circulation due to enzymatic (e.g., nucleases, such as deoxyribonucleases, and RNAse, such as ribonucleases) and chemical (e.g., oxidation, hydrolysis, deamidation, depurination, and strand cleavage) degradation. Therefore, chemical modifications are usually necessary to improve NA stability. Currently, messenger RNA (mRNA) vaccines have become the frontrunners in fighting coronavirus (COVID-19). However, mRNAs alone are prone to nuclease degradation and phosphate backbone hydrolysis through the intramolecular attack of the 2′-hydroxyl group in physiological fluids [13], which is responsible for the short half-life and low efficiency of the mRNA therapeutics due to incompatibility with nuclease, high molecular weight, high negativity, and their hydrophilic and acid-labile nature [14]. Therefore, lipid nanoparticles (LNPs) are employed in COVID-19 mRNA vaccines. A key disadvantage of the approved COVID-19 mRNA-LNP vaccines is the need to be kept under (ultra) cold storage conditions [15], and the stability of mRNA vaccines during storage, handling, and shipping at ambient temperatures is a primary concern [16,17]. As discussed earlier, mRNA could also undergo hydrolytic degradation during storage, handling, and shipping at ambient temperatures. Thus, DDSs, such as LNP, act as a shield for mRNA to prevent degradation and assure its potency.

In the case of antisense oligonucleotides (ASOs), modifications are usually made to the 2′-carbon of the sugar ring or phosphodiester bond. Phosphorothioate modification provides key protection from nucleases and extends the half-life with better stability. A similar phosphonoacetate modification is possible at the ASO backbone, which is totally resistant to nuclease degradation. Another approach to enhance ASO stability is the ribose sugar modification at the 2′-position of the ring, e.g., locked nucleic acids (LNAs) [18,19]. However, in phosphorodiamidate morpholino oligomers (PMOs), ribose sugars are exchanged with phosphodiester bonds and morpholino groups (e.g., casimersen), sometimes referred to as splice switching oligonucleotides (SSOs). For example, nusinersen and eteplirsen are inclusion- and skipping-type SSOs, respectively. In the case of peptide nucleic acids (PNAs), the ribose-phosphate backbone is exchanged with a polyamide backbone [20,21]. In addition, short interfering RNA (siRNA) chemical modifications at various sites, such as phosphate, nucleobases, ribose, poly-2-O-(2,4-dini-trophenyl)-RNA, and oxygen ring replacement with a sulfur group, exhibit enhanced resistance to RNase with better stability. Fluorine, sugar, methoxy, or deoxy modifications at 2′ positions of the ring are other approaches to improve siRNA serum stability [22,23,24]. On the other hand, targeting the liver is both a major advantage and disadvantage (e.g., immunotoxicity, immunogenesis, degradation of LNP against the harsh GIT conditions) of NA therapeutics [25]. Due to their different particle size and lipid composition, nanocarriers have organ-specific targeting. LNP is likely delivering the drug to the liver. Rizvi et al. [26] measured the organ distribution of protein activity produced by firefly luciferase encoding mRNA-LNP (Luc mRNA-LNP) and found that robust luciferase expression was detected in the liver. Yang et al. [27] used the lipopolyplex (LPP) nanoparticles, formulated by SW-01(a positively charged cationic compound), ionized and non-ionized lipids, carrying with mRNA encoding luciferase which was evaluated for biodistribution pattern. After intramuscular injection (i.m.) into the hind legs of mice, a strong luciferase expression was noticed at the injection site (muscle) but not in the liver.

In addition, the ASOs are modified with PS-linkages (e.g., Kynamro, Tegsedi), and siRNA (e.g., Oxlumo) are well targeted to hepatic delivery. Synthetic therapeutic oligonucleotides (STOs) could control numerous intracellular and extracellular obstacles to interact with their biological RNA targets inside cells. STOs depend on passive exchanges of phosphorothioate (PS) oligonucleotides with cell-surface and plasma proteins to endorse delivery to the kidney and liver. PS-mediated drug delivery was also useful in delivering NA therapeutics (e.g., Spinraza) to CNS tissues [28].

Additionally, gene-based therapies present exclusive challenges due to several factors, including membrane fluidity and permeability and multiple and complex antigen epitopes. Aggregation, oxidation, deamidation, hydrolysis, and adsorption of gene therapies are frequent pathways responsible for degradation. During deamidation, a succinimidyl intermediate is formed due to nucleophilic attack by the adjacent amide over the amide group of asparagine, and the succinimidyl intermediate is hydrolyzed to isoaspartic and aspartic acids [29]. Thus, this causes instabilities in cell-based therapeutics [30], which may lead to the loss of therapeutic efficacy and produce severe immunogenic responses in patients [31]. Therefore, an astute consideration of the degradation processes and triggering factors and the selection of suitable excipients are crucial to protect NA therapeutics against destabilization.

The challenges involved in the successful formulation of NA therapeutics include a variety of physicochemical degradation pathways. The mechanisms of physicochemical degradation with various environmental factors influencing NA stability have been broadly studied. Physical instability is due to exposure to different temperatures, pH, buffer concentrations, presence of oxygen, ultraviolet light, transition metal cations (e.g., copper, ferrous, zinc, magnesium, and nickel), and various stress conditions. For example, uracil is the deamidate product of cytosine, and the process is 100-fold quicker in single-stranded NAs. At higher temperatures and above pH 7, depurination is observed to be sequence-independent. Metal contamination is a significant factor in the degradation of NAs. During the manufacture of NAs, trace amounts of metals present in various raw materials can be established. Transition metal cations have been found to form metal-base pairs by chelation and initiate the breakage of NA strands. Chelation occurs between the NA strands by connecting the cytosines and two adenines, or mixtures thereof [32,33]. Among the transition metals present, copper and ferrous metal ions seem to be strong degradation catalysts. For example, DNA degradation is observed via the intermediate formation of the DNA-copper-hydroperoxide complex by copper ions and ferrous ions, which have been found to be responsible for the degradation of calf thymus DNA due to molecular oxygen [34]. Chemical instability is prevalently caused by hydrolysis, oxidation, depyrimidination, and deamination [35]. In hydrolysis, phosphoester and N-glycosidic bonds are more prone to hydrolytic cleavage. Nucleophilic phosphodiester cleavage is caused by either inter- or intramolecular reactions. Oxidative degradation primarily involves reactive oxygen species (ROS) reactions in which Fenton-type processes are the most frequent origin of ROS. In addition, the formation of covalent intrastrand purine dimers [36] and oxidation through free radicals are considered chief degradative processes for NA therapeutics [37]. Irradiation with NAs was found to generate hydroxyl free radicals that also caused damage [38].

As specified, NA therapeutics are usually formulated as freeze-dried powders, similar to many protein drugs [39,40]. NAs are generally more stable in a freeze-dried (lyophilized) form than in a liquid state. In the freeze-dried form, many of these degradation pathways may be avoided or retarded. Therefore, freeze-drying is advised to be a practical method to sort out stability problems during long-term storage [41]. siRNA has been administered as a dry powder inhalation (DPI) to facilitate the delivery of drugs in lung therapy [42,43]. In addition, Gennova biopharmaceuticals-based mRNA HGCO19 vaccine will be in a lyophilized form. Recently, the approved tozinameran (BNT162b2, Pfizer/BioNTech) and elasomeran (mRNA-1273, Moderna) mRNA vaccines have been considering sucrose as a lyoprotectant [44] due to the surprising loss of mRNA stability and delivery efficiency following the lyophilization of LNP [45,46]. Therefore, it is necessary to optimize freeze-drying parameters and choose a suitable lyoprotectant to achieve a stable product.

### 2.2. NA Therapeutics Delivery

The use of NA therapeutics has climbed significantly in the last decade, but there are still clinical challenges, such as poor pharmacokinetics and pharmacodynamics [47,48,49]. Incompetent delivery to target organs is a key hurdle preventing the prevalent usage of NA therapeutics (Figure 1). The design of delivery vehicles with particular features renders them stable, efficient, and safe in transfection.

The delivery of NA therapeutics can be influenced by their attributes, such as a negative charge, hydrophilicity, and susceptibility to enzyme degradation [50]. In addition, off-target side effects must be cautiously monitored. Therefore, these issues need to be addressed for the timely development of smart NA formulation. The most commonly engaged strategies to boost NA delivery include chemical, ribose sugar, nucleobases, backbone, and terminal modifications with cell-penetrating moieties. The approaches which have been developed most recently include liposomes, lipoplexes, NPs, DNA cages, DNA nanostructures (framework nucleic acids) [51], microspheres, exosomes [11], gene therapy, spherical NA, red blood cells, biological solids, stimuli-responsive nanotechnology, polyplexes, extracellular vesicles (natural and engineered), micelleplexes, heteroduplex oligonucleotides, niosomes (unilamellar and multilamellar), carbon nanotubes, carbon nanodots, and aptamers [52,53,54,55,56,57,58,59,60,61,62,63]. Furthermore, Maurer et al. developed magnetic hybrid niosomes (iron-oxide NPs) for siRNA delivery to treat breast cancer [64,65]. All these NA delivery systems have contributed to overcoming several challenges related to NA therapeutics, which include protecting them from degradation and avoiding renal excretion, thereby improving the safety profile.

NP-based drug delivery represents a highly adaptable platform for a variety of therapeutics [66,67]. This includes lipid-based NPs (e.g., liposomes, cubosomes, ionizable, and solid LNPs), polymeric NPs (e.g., natural and synthetic polymers), metal NPs (e.g., gold, silver, and iron), gold NPs (e.g., spherical and nonspherical (such as nanostars, nanorods, and nanocubes)), porous NPs (e.g., porous silicon and mesoporous silica NPs), and metal-organic frameworks (e.g., NU-100) [68,69,70]. The most frequently employed biodegradable natural polymers are alginate, hyaluronic acids, and chitosan. The current use of synthetic polymers over natural polymers has received noticeably more attention due to their better mechanical and reproducible properties, i.e., dendrimers (e.g., poly-(β-amino ester) (PβAE), poly-(L-lysine) (PLL), and polyamidoamine (PAMAM)), PLGA (polylactic-co-glycolic acid), and PEIs (polyethylene imines) [71,72].

Novel DDSs could enhance the solubility, bioavailability, safety, and PK profiles of systemically administered drugs, leading to enhanced therapeutic efficacy [73]. Therefore, formulation scientists are paying attention to easy and smart DDSs. The current scenario shows rapid commercialization and increasing popularity of nanomedicine dominance over the other DDSs [74,75,76,77]. However, liposomes face parallel challenges, including restrictions from the risk of causing immune responses and biodistribution [78]. Due to the suitability of liposomes to both hydrophilic and lipophilic drug candidates, they are used as carriers of choice for various therapeutics [79]. The leading pathway for siRNA therapeutics delivery to the liver was found to be N-acetyl galactosamine (GalNAc), which has proven to be long-acting, therefore, improving the safety profile of the NA therapeutics [80]. In addition, lipid-based carriers (e.g., cationic lipids) [81], polyplexes (many mRNA-based drugs under clinical trials), multivalent cationic non-viral vectors, and polymeric vehicles [82,83,84] represent the most commonly preferred alternative to viral vectors in gene therapy [85,86,87]. Viral vectors with antigen-encoding RNA in place of viral genes are prepared by genetic modification of viruses and are effective delivery vehicles. Viral vectors are categorized as replicating and non-replicating vectors [88]. Various RNA viruses, such as adenovirus, picornavirus, flavivirus, and alphavirus, have been used as viral vectors for mRNA delivery. Viral vectors have several limitations, including host genome integration (genotoxicity) and allergic reactions. Therefore, viral vectors have been replaced by non-viral vectors. Non-viral vectors can be classified as hybrid, polymer-based, and lipid-based [36]. They have several benefits over viral vectors, such as ease of production, multi-dosing capabilities, lesser toxicity, and the lack of immunogenicity [89,90]. Therefore, non-viral vectors could be designed with multiple components to overcome physiological barriers [91].

In 1995, the liposome Doxil emerged as the first NP therapeutic. In 2005, human albumin was employed in NP formulations (e.g., Abraxane). Recently, in 2017 [92], the FDA approved the first gene therapies (e.g., Kymriah), which were prepared from the white blood cells of the patients. The great commercial achievement of these DDSs has fascinated many professionals in the field [93]. DDSs play a crucial role in biopharmaceutical formulations, including proteins, monoclonal antibodies, and, recently, NAs. The advanced DDSs promote the high quality and efficacy of the drugs and extend the shelf life of these new molecular entities. Among the approved NA therapeutics, many are delivered as novel DDSs. Patisiran (Onpattro^®^) is the first commercially available LNP formulation delivered in liposome vesicles. In addition, some gene therapies are associated with adenovirus vectors (e.g., voretigene neparvovecrzyl and onasemnogene abeparvovec-xioi), herpes simplex virus vectors (e.g., talimogene laherparepvec), and gamma retroviral vectors (e.g., strimvelis) [94,95,96]. Concerning other gene therapies, adeno-associated virus (AAV) has emerged as the principal vector due to the sustainability of the viral genome and its lack of pathogenicity. AAV vectors have special features, such as requiring a helper virus for replication and avoiding pathogenicity. They also have a low tendency for gene integration and, therefore, avoid genotoxicity [97]. The FDA approved Luxturna^®^ as AAV2-based and Zolgensma^®^ as AAV9-based gene therapy, and many more AAV-based gene therapies are under clinical trials [98].

Recently, numerous RNA and DNA vaccines have entered the clinical stages. Among them, mRNA vaccines have become therapeutic targets of interest in many severe diseases and disorders. The safe and efficient delivery of therapeutic mRNAs is one of the key challenges for their wide implementation in humans. mRNAs have concerns such as sensitivity to catalytic hydrolysis, intracellular delivery, and high instability under certain physiological conditions [99]. Recently, developments in mRNA vaccines have been made through their formulation with LNP, which not only provides enhanced delivery and protection but also performs the role of an adjuvant in vaccine reactogenicity [100,101], i.e., the LNP-based delivery of mRNA vaccines against influenza and Zika [102]. In addition, nanoscale delivery platforms (e.g., lipid-derived NPs, polymeric NPs, polymer-lipid hybrid NPs, metal NPs, and peptide complexes) have prolonged the viability of mRNA-based therapeutics, which permit their promising application in protein replacement therapy, genome editing, and cancer immunotherapy [103]. Currently, a major vaccine development platform is advancing with self-assembling drug delivery vehicles, such as cationic monovalent lipids (e.g., N-[1-(2,3-dioleyloxy)propyl]-N,N,N-trimethylammonium chloride (DOTMA), 1,2-dioleoyl-3-tri-methylammonium propane (DOTAP), cationic multivalent lipids (e.g., 2,3-dioleyloxy-N-(2(sperminecarboxaminino)ethyl)-N,N-dimethyl-1-propanaminium trifluroacetate (DOSPA), dioctadecylamidoglycylspermine (DOGS), neutral lipids (e.g., 1,2-dioleoyl-snglycero-3-phosphoethanolamine (DOPE), dioleoyl phosphatidylcholine (DOPC), and polymeric (e.g., poly(beta-amino esters)), poly(poly-polyesters), poly(colactic glycolic acid), and dioleoyl(DOPC). This involves the use of mRNA-lipid-derived NPs as principal components. In addition to cationic and neutral lipids, anionic lipids have been useful in gene delivery (e.g., dioleoylphosphatidic acid (DOPA), dihexadecyl phosphate (DHPG), dioleoylphosphatidylglycerol (DOPG), and dioleoylphosphatidylserine (DOPS) [104,105,106,107].

In particular, LNP-based therapeutics have been proven to be highly biocompatible over polymeric and metal-based delivery systems. It is well-reported that the practice of polymer and metal-based NPs may exert adverse effects [108]. Many vaccine candidates are under clinical trials (e.g., COVAC1, CvnCoV, and LUNAR^®^-COV-19) [109,110,111,112]. Recently, the FDA approved the outstanding vaccine candidate’s tozinameran and elasomeran initially under emergency use authorization (EUA) in late 2020 and subsequently granted full approval in mid-2021 for the control of the worldwide COVID-19 pandemic. This has proven to be a breakthrough for the global health emergency of COVID-19. In collaboration with the National Institute of Allergy and Infectious Diseases, Moderna developed the lipid NP-encapsulated mRNA-based vaccine named elasomeran [113]. Simultaneously, Pfizer’s mRNA vaccine candidate tozinameran satisfied the entire preliminary efficacy endpoints and was granted the Medicines and Healthcare Products Regulatory Agency (MHRA) approval. The tozinameran vaccine is supplied as a frozen lipid NP suspension. The development of the vaccines was complex, and the Pfizer vaccine (tozinameran) must be stored at −80 to −60 °C, while the Moderna vaccine (elasomeran) must be stored at −25 to −15 °C, making them difficult to handle worldwide, especially in tropical regions.

Therefore, merging NA therapeutics with suitable novel DDSs has become imperative for improving the efficacy with targeted drug delivery and potentially lowering the dose regimens, which may equally reduce the cost.

## 3. Commercially Approved NA Therapeutics

NA therapeutics can be generally categorized based on the origin and size of the drug. This includes oligonucleotides, ASOs, siRNA, gene therapy, and mRNA vaccines (Table 1) [114]. Oligonucleotides are short DNA or RNA molecules. ASOs are a short single-stranded DNA. The siRNAs are small, double-stranded RNAs with each strand being 20–30 nucleotides. mRNA molecules are composed of thousands of nucleotides with high molecular weights. The modes of action of NAs are different from that of other drugs. NAs are directly delivered to target cells and tissues. Due to their high molecular weight and highly hydrophilic nature, they do not penetrate cells easily and are prone to degradation. Therefore, NAs need high-quality formulations with suitable DDSs to protect them from degradation and to ensure delivery to targeted cells or tissues [115,116]. NA therapeutic formulation development is especially challenging in terms of physical, chemical, and conformational instability. Therefore, an appropriate choice of excipients and drug delivery could lead to high-quality and stable NA therapeutics.

Usually, excipients are the key components of a formulation, of which the active drugs comprise only a tiny proportion of the total composition [117]. The key functions of excipients are to improve the safety, stability, and efficacy of therapeutics. A few excipients may be added to formulations to provide tonicity to minimize pain upon injection or to target the easy delivery in the body upon administration (i.e., buffers for pH control, protectants for higher stability, bulking agents for freeze-drying, a surfactant for adsorption control, and salts for osmolality adjustment) [118]. Excipients are comprised of integral components of any formulation. As noted by a model excipient, it is chemically compatible, safe, stable, economical, and multifunctional [119]. In the case of NA formulations, they not only function to regulate shifts in pH but can also stabilize NAs by a variety of mechanisms [92].

The ability of excipients to stabilize therapeutic NAs is notable. The names of excipients used in approved NA therapeutics are shown in Table 2 [120], which summarizes the common excipients included in NA therapeutics for each functional category. The information is briefly tabulated as the commercial name, APIs, dosage form, therapeutic class, excipient compositions, strength, dosage, pH range, administration route, storage condition, and date of approval by the governing authorities (e.g., FDA and EMA).

NA therapeutics are manufactured in different dosage forms (e.g., solution, suspension, freeze-dried, or vector-like liposome/lipids, and NPs). Excipients can play key roles when employed in optimized concentrations [121]. The compositions of approved NA therapeutics are reported, and the relevant information has been gathered from accredited sources, such as DrugBank.com (10 December 2022) and FDA labels (www.fda.gov, 12 December 2022).

The selection of excipients for any formulation requires the verification of some basic factors, such as the API concentration, dosage form (liquid or freeze-dried), administration route, and compatibility. In the case of parenteral formulations, the choice of excipients is fairly limited. Therefore, the proper selection and optimization of excipients are crucial factors for protecting against physicochemical instabilities.

The pH of the formulation has a strong impact on the NA stability. The degradation pathway usually involves a two-step process, such as β-elimination and depurination, which are acid- and base-catalyzed, respectively. A cytosine-deaminated product by sodium bisulfite was detected in acidic buffers (i.e., pH < 6.0) [122]. At an alkaline pH (i.e., pH > 13), the bisulfite adduct is transformed into uracil through bisulfite elimination, whereas an N-glycoside bond cleavage is responsible for the conversion of the pyrimidine-sulfite adduct into a basic site [123]. Therefore, buffers are necessary to maintain the pH at which the specific NA has maximal stability. A pH in a physiological range is more suitable for the easy administration of a drug. It was recommended that a low buffer concentration would minimize the risk of a pH shift [124]. The approved NA therapeutics have specific pH values, i.e., patisiran, viltolarsen, vutrisiran, and lumasiran sodium at pH 7.0; voretigene neparvovecrzyl at pH 7.3; nusinersen at pH 7.2; mipomersen sodium, eteplirsen, inotersen, casimersen, and golodirsen at pH 7.5; volanesorsen sodium at pH 8.0.

The selection of any suitable buffer depends on compatibility with the NA and its formulation excipients. Occasionally, the absence of buffers in NA formulations might negatively impact the quality profile of formulations. In 2015, Garidel et al. tried a successful buffer-free strategy in freeze-dried protein formulations [125]. This might be an important consideration for NA drugs.

The majority of NA therapeutics, including pegaptanib, nusinersen, eteplirsen, vutrisiran, and golodirsen, use monobasic and dibasic sodium phosphate as the buffer. On the other hand, eteplirsen and golodirsen have used monobasic potassium phosphate as the buffering agent to prevent a pH shift. In addition, they also contain sodium chloride, sodium citrate, and/or potassium chloride in optimal concentration ranges as tonicifiers and may protect them from pH oscillations during storage [126].

Primarily, for pH adjustment, mipomersen sodium, inotersen, volanesorsen sodium, defibrotide sodium, inclisiran sodium, casimersen, vutrisiran, and viltolarsen contain sodium hydroxide and hydrochloric acid as excipients. Defibrotide sodium and viltolarsen additionally contain sodium citrate and sodium chloride as tonicifiers, respectively.

In contrast, few NA therapeutics, such as siRNA-based patisiran and both mRNA-based vaccines, are versatile in terms of excipients. All three products are composed of four lipids showing only differences in the storage conditions. For example, patisiran has diverse excipients, such as cholesterol, (6Z, 9Z, 28Z, 31Z)-heptatriaconta-6,9,28,31 tetraen-19-yl-4-(dimethylamino)-butanoate-(DLin-MC3-DMA), functioning as a conjugation for delivery [127], and 1,2-distearoyl-sn-glycero-3-phosphocholine (DSPC) as a helper lipid to protect NPs from aggregation, with diffusible α-(3′-{[1,2-di(myristyloxy)propanoxy]-carbonylamino}propyl)-ω-methoxy, polyoxyethylene (PEG 2000 C-DMG). In addition, all these vaccines play key roles in efficient delivery and enhancing active pharmaceutical ingredients. The carriers may also regulate the safety profile of these drugs to a great extent, which helps to improve the ultimate drug performance and add a valuable contribution to safety, as well as targeted delivery.

In addition, the latest COVID-19 mRNA vaccines contain cholesterol, the helper lipid DSPC, and the diffusible PEG-lipid ((2-[(polyethylene glycol)-2000]-N,N-ditetradecylacetamide, PEG 2000-DMA in tozinameran, or PEG 2000-DMG, 1,2-dimyristoyl-rac-glycero3-methoxypolyethylene glycol-2000 in elasomeran)) [15]. Cholesterol is responsible for lipid membrane fluidity. PEG chain-linked phospholipids function as excipients that furnish a hydrophilic layer and prolong the half-life of mRNA. An optimized concentration of sucrose and sodium phosphate is used to stabilize liposomal NPs during shipping [126]. The ionizable lipids SM-102 and ALC-0315 are employed in the LNP formulations of elasomeran and tozinameran, respectively [15]. Tozinameran and elasomeran contain formulations of ionizable:cholesterol:neutral lipid:PEGylated lipid at molar ratios of 46.3:42.7:9.4:1.6 mol% and 50:38.5:10:1.5 mol%, respectively, along with a lipid:mRNA ratio of 0.05 (*w*/*w*). In the mRNA vaccine formulation, sucrose functions as a cryoprotectant during freeze–thaw cycles [128,129]. Similarly, CVnCoV (CureVac/BAYER), currently in phase 2b/3 clinical trials, has molar ratios similar to those of elasomeran [14,130,131,132,133]. Currently, several LNP-based mRNA vaccines are in the clinical pipeline to prevent COVID-19, such as ARCT-021 by Arcturus Therapeutics [134], ARCoV by Walvax Biotechnology/Suzhou Abogen Biosciences [135], DS-5670 by Daiichi Sankyo/the University of Tokyo [136], and LNP-nCoV RNA by Imperial College London [137]. In addition, LNP-based mRNA vaccines, such as mRNA-1325 and mRNA-1893 for Zika virus, mRNA-1944 for chikungunya, CV7202 mRNA for rabies, VAL-506440/H10N8 antigen mRNA, and VAL-339851/H7N9 antigen mRNA for influenza are under Phase 1 clinical trial [138]. LNPs composed of biodegradable ionizable lipids could be a promising next-generation delivery system.

A surfactant can stabilize NA by increasing its solubility while minimizing interfacial and nonspecific interactions. The voretigene neparvovecrzyl gene therapy has used poloxamer 188 as a surfactant at optimal concentrations. In addition, patisiran, tozinameran, and elasomeran have PEG 2000 C-DMG, ALC-0159, and PEG-DMG, respectively.

Talimogene laherparepvec contains sorbitol as a stabilizer with sodium dihydrogen and disodium hydrogen phosphate dihydrate as buffering agents. In addition to buffer, nusinersen contains calcium chloride dihydrate with magnesium chloride hexahydrate as complexing agents, which may chelate trace amounts of transition metals present in the buffer to prevent degradation [139]. However, the alipogene tiparvovec injection has used potassium dihydrogen phosphate, disodium phosphate, sucrose, potassium, and sodium chloride as the excipients for formulation. Unfortunately, the sponsor allowed the approval to expire due to the poor commercial absorption of the drug in the market in 2017 [140,141].

**Figure 2:**
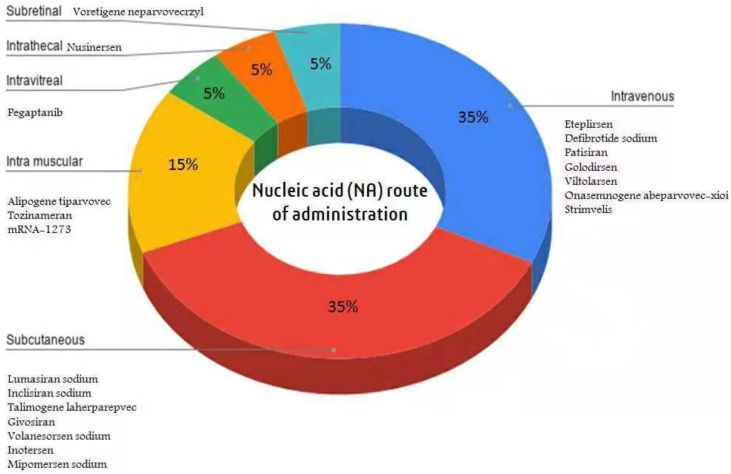
Route of administration allocation for approved NA therapeutics.

In addition to the drug delivery approach, the selection of suitable administration routes is also a significant factor in ensuring efficient and safe drug delivery. Primarily, NA therapeutics are designed for parenteral administration because they are often hard to administer via non-parenteral routes [142]. Therefore, NAs must be formulated as a stable liquid or freeze-dried powder to deliver these drugs safely and efficiently to their target site [143]. To target drug delivery sites, NAs need to be carefully tailored. The novel trends are set to subcutaneous (SC) administration, and the percentage of NA therapeutics approved for SC administration has risen gradually. Among the approved NA therapeutics, 35% of each have been intended for SC and IV administration (Figure 2). Recently, the approved mRNA vaccines for COVID-19 have been administered intramuscularly (IM). NA formulations with a high strength (i.e., >80 mg/mL) are more frequently delivered subcutaneously. Therefore, the NA concentrations in the formulations may depend on the therapeutic effect and the selected administration route for drug delivery. For example, nusinersen (2.4 mg/mL) was administered via the intrathecal route (IT), pegaptanib (0.4 mg/mL) via the intravitreal route (IVI), and mipomersen sodium (200 mg/mL) via the SC route.

It is important to include tonicifiers in NA therapeutics to maintain isotonicity for superior parenteral administration. Most NA therapeutics use potassium chloride and/or sodium chloride as tonicifying agents; however, additional excipients could be added to advance the quality of the therapeutics.

Another crucial aspect is the storage condition. Long-term storage can affect the physicochemical stabilities of NA therapeutics. Sulfur substitutions on the phosphate backbone could easily be exchanged back to dissolved oxygen at elevated temperatures, making the products more prone to nuclease attack. The majority of NA therapeutics are stored at 2–8 °C. The exceptions include voretigene neparvovecrzyl, stored at ≤−65 °C, alipogene tiparvovec stored at −25 °C to −15 °C, defibrotide sodium stored at 20–25 °C, and givosiran, vutrisiran, and lumasiran stored at 2–25 °C. The new group mRNA vaccines tozinameran (Pfizer-BioNTech) and elasomeran (Moderna) are recommended to be stored at −80 °C to −60 °C and −25 °C to −15 °C, respectively. A recent study concluded that mRNA vaccines are stored at a low temperature to avoid or slow down mRNA degradation by hydrolysis during storage [128].

## 4. Conclusions and Future Perspectives

Over the past decade, the impact of NA therapeutics has been facilitated by breakthroughs associated with high-end manufacturing and drug delivery in the field of pharmaceuticals. Although excellent growth has been made in NA delivery, including intracellular delivery, several challenges and requirements remain.

This article also discusses the prime considerations pertaining to novel excipients and analyzes their function and rationale in NA therapeutics. To date, 23 NA therapeutics have been approved; however, the publicly accessible awareness of NA therapeutics is extremely limited. Therefore, we recommend that scientists carefully monitor the stability during all stages of NA therapeutics development. Simultaneously, the trend of novel excipients and SC administration is also garnering attention, predominantly in terms of highly concentrated NA therapeutics. In addition, it is important to monitor deficiencies caused by the administration of various NA therapeutics. For example, vutrisiran has caused vitamin-A deficiency in patients. Therefore, the provision of vitamin-A supplementation is a primary concern during the therapeutic application of vutrisiran. In future publications, we aim to discuss the adverse events and the precautionary actions taken during NA therapeutics therapy.

Looking at the current scenario, the development of NAs as therapeutics focuses on long-term stability and the efficiency of DDSs, such as liposomes, NPs, microspheres, or gene therapy. However, there is a need to address several challenges posed by DDSs, such as manufacturing complexity, cost-effectiveness, and safety. In addition, self-amplifying RNA would be a next-generation vaccine platform but requires smart drug delivery vehicles to maintain the long-term stability and efficiency of the drug. In the near future, developing long-acting DDSs to improve the PK and enhance the targeting efficiency at cellular and tissue sites is critical. Formulation scientists could merge advanced techniques, like artificial intelligence and machine learning, with the DDSs to make them more intelligent and potentially more affordable, and easier to use for patients. This continuous advancement presents the hope that remedies for rare or currently untreatable diseases will soon be possible and affordable.

## Figures and Tables

**Table 1:** Classification of approved nucleic acid (NA) therapeutics.

Biologic Classification	Drug Name (Brand Name)	Subunits	Mol Formula	Target	Indication	Drug Delivery	Approving Agency	Approval Year
Oligonucleotides	Pegaptanib (Macugen)	27	C_294_H_342_F_13_N_107_Na_28_O_188_P_28_[C_2_H_4_O]_2_n	Selectively binds to VEGF165	Neovascular (wet) age-related macular degeneration.	Naked	FDA	2004
	Mipomersen (Kynamro)	20	C_230_H_324_N_67_O_122_P_19_S_19_	mRNA of apoB-100	Familial hypercholesterolemia	Naked	FDA	2013
	Defibrotide (Defitelio)	-	C_20_H_21_N_4_O_6_P	Adenosine receptors A1, A2a, A2b	Severe hepatic veno-occlusive disease	Naked	FDA	2016
Antisense oligonucleotides	Fomivirsen (Vitravene)	21	C_204_H_243_N_63_O_114_P_20_S_20_Na_20_	30 kDa and 54 kDa immediate-early protein 2	Cytomegalovirus (CMV) retinitis in patients with AIDS	Naked	FDA	1998
	Eteplirsen (Exondys 51)	30	C_364_H_569_N_177_O_122_P_30_	Forcing the exclusion of exon 51 from the mature DMD mRNA	Duchenne muscular dystrophy	Naked	FDA	2016
	Nusinersen (Spinraza)	18	C_234_H_323_N_61_O_128_P_17_S_17_Na_17_	Survival motor neuron-2 protein	Spinal muscular atrophy	Naked	FDA	2016
	Inotersen (Tegsedi)	20	C_230_H_318_N_69_O_121_P_19_S_19_	Transthyretin mRNA	Polyneuropathy	Naked	FDA	2018
	Volanesorsen (Waylivra)	-	C_230_H_320_N_63_O_125_P_19_S_19_	Binds to apo C-III mRNA	Familial chylomicronemia syndrome	Naked	EMA	2019
	Golodirsen (Vyondys 53)	25	C_305_H_481_N_138_O_112_P_25_	Dystrophin	Duchenne muscular dystrophy	Naked	FDA	2019
	Viltolarsen (VILTEPSO)	21	C_244_H_381_N_113_O_88_P_20_	DMD gene (exon 53 viltolarsen target site)	Duchenne muscular dystrophy	Naked	FDA	2020
	Casimersen (Amondys 45)	20	C_268_H_424_N_124_O_95_P_22_	DMD gene (exon 45)	Duchenne muscular dystrophy	Naked	FDA	2021
Short interfering RNA (siRNA)	Patisiran (Onpattro)	21	C_412_H_480_N_148_Na_40_O_290_P_40_	Transthyretin mRNA	Polyneuropathy	LNP	FDA	2018
	Givosiran (Givlaari)	21	C_524_H_694_F_16_N_173_O_316_P_43_S_6_	ALAS1 mRNA	Acute hepatic porphyria	N-acetylgalactosamine	FDA	2019
	Lumasiran (Oxlumo)	-	C_530_H_669_F_10_N_173_O_320_P_43_S_6_Na_43_	hydroxyacid oxidase-1 (HAO1) mRNA in hepatocytes	Primary hyperoxaluria type 1	N-acetylgalactosamine	FDA	2020
	Inclisiran (Leqvio)	-	C_520_H_679_ F_21_N_175_O_309_P_43_S_6_	Inhibit hepatic translation proprotein convertase subtilisin-Kexin type 9 (PCSK9)	Primary hypercholesterolemia	N-acetylgalactosamine	EMA/FDA	2020/2021
	AMVUTTRA (Vutrisiran)		C_530_H_715_F_9_N_171_O_323_P_43_S_6_	Transthyretin mRNA	Amyloidogenic Transthyretin Amyloidosis	Naked	FDA	2022
Gene therapy	Voretigene neparvovecrzyl (Luxturna)	-	-	Human retinal pigment epithelial 65 kDa protein (RPE65) encoded gene	Retinal dystrophy	Adeno-associated virus vector	FDA/EMA	2017/2018
	Onasemnogene abeparvovec-xioi (Zolgensma)	-	-	Gene encoding copy delivery to the human SMN protein	Spinal muscular atrophy	Adeno-associated virus	FDA	2019
	Alipogene tiparvovec (Glybera)	-	-	-	Severe pancreatitis	Naked	EMA	2012
	Talimogene laherparepvec (Imlygic)	-	-	For the production of immune response stimulatory protein, human GM-CSF	In recurrent melanoma	Herpes simplex virus 1	FDA/EMA	2015
	Strimvelis	-	-	Activate ADA enzyme	Adenosine deaminase-severe combined immunodeficiency (ADA-SCID)	Gamma retroviral vector	EMA	2016
mRNA vaccines	Tozinameran (Comirnaty) (BNT162b2)	4284	-	SARS-CoV-2S glycoprotein	COVID-19	LNP	FDA/EMA	2021
	Elasomeran (Spikevax) (mRNA-1273)	4284	-	SARS-CoV-2S antigen	COVID-19	LNP	FDA/EMA	2021

**Table 2:** In brief therapeutic information of approved nucleic acid (NA) therapeutics.

API	Dosage Form	Excipients	Strength	Dosage	pH Range	Route of Administration
Pegaptanib (0.3 mg)	Injection/solution	Dibasic sodium phosphate heptahydrate, monobasic sodium phosphate monohydrate, sodium chloride sodium hydroxide, and hydrochloric acid	0.4 mg/mL, 3.47 mg/mL	0.3 mg/90 µL	6.0 to 7.0	IVI
Mipomersen sodium (200 mg)	Injection/solution	Hydrochloric acid and sodium hydroxide	200 mg/mL	200 mg/mL solution	7.5 to 8.5	SC
Eteplirsen (50 mg)	Injection/solution	0.2 mg potassium phosphate monobasic, 0.2 mg potassium chloride, 8 mg sodium chloride, and 1.14 mg sodium phosphate dibasic, anhydrous hydrochloric acid, and sodium hydroxide	50 mg/mL	100 mg/2 mL, 500 mg/10 mL	7.5	IV
Nusinersen (2.4 mg)	Injection/solution	0.16 mg magnesium chloride hexahydrate USP, 0.22 mg potassium chloride USP, 0.21 mg calcium chloride dihydrate USP, 8.77 mg sodium chloride USP, 0.10 mg sodium phosphate dibasic anhydrous USP, 0.05 mg sodium phosphate monobasic dihydrate USP, hydrochloric acid and sodium hydroxide	2.4 mg/mL	12 mg/5 mL	~7.2	IT
Defibrotide sodium (80 mg)	Injection/solution	10 mg sodium citrate USP, hydrochloric acid, and sodium hydroxide	80 mg/mL	200 mg/2.5 mL	6.8–7.8	IV
Inotersen (284 mg)	Injection/solution	Phosphate buffer, hydrochloric acid, and sodium hydroxide	284 mg/1.5 mL	284 mg/1.5 mL	7.5 to 8.5	SC
Patisiran (2.0 mg)	Injection/liposome	13.0 mg (6Z,9Z,28Z,31Z)-heptatriaconta-6,9,28,31 tetraen-19-yl-4-(dimethylamino) butanoate (DLin-MC3-DMA), 3.3 mg 1,2-distearoyl-sn-glycero-3-phosphocholine (DSPC), 6.2 mg cholesterol USP, 1.6 mg α-(3′-{[1,2-di(myristyloxy)propanoxy] carbonylamino}propyl)-ω-methoxy, polyoxyethylene (PEG 2000 C-DMG), 0.2 mg potassium phosphate monobasic anhydrous NF, 2.3 mg sodium phosphate dibasic heptahydrate USP, and 8.8 mg sodium chloride USP	2 mg/mL	10 mg/5 mL	~7.0	IV
Givosiran (189 mg)	Injection/solution	Water for injection	189 mg/mL	189 mg/mL	-	SC
Volanesorsen sodium (200 mg)	Solution	Sodium hydroxide and hydrochloric acid	200 mg of Volanesorsen sodium/mL	285 mg of Volanesorsen/1.5 ml	8.0	SC
Golodirsen (50 mg)	Injection	0.2 mg potassium phosphate monobasic, 0.2 mg potassium chloride, 8 mg sodium chloride, and 1.14 mg sodium phosphate dibasic anhydrous, sodium hydroxide, and hydrochloric acid	50 mg/mL	100 mg/2 mL	7.5	IV
Viltolarsen (50 mg)	Injection/solution	9 mg (0.9%) sodium chloride, sodium hydroxide, and hydrochloric acid	50 mg/mL	250 mg/5 mL	7.0 to 7.5	IV
Casimersen (50 mg)	Injection/solution	0.2 mg potassium chloride, 0.2 mg potassium phosphate monobasic, 8 mg sodium chloride, and 1.14 mg sodium phosphate dibasic	50 mg/mL	100 mg/2 mL	7.5	IV
Voretigene neparvovecrzyl	Solution/suspension	10 mM sodium phosphate, 180 mM sodium chloride, and 0.001% poloxamer 188	5 × 10^12^ vg/mL	0.3 mL, 0.5 mL in 2 mL	7.3	IOI
Onasemnogene abeparvovec-xioi	Suspension	Onasemnogene abeparvovec (20000000000000 1/1 mL) + Onasemnogene abeparvovec (20000000000000 1/1 mL) + isopropyl alcohol (0.7 mL/1 mL)	2.0 × 10^13^ vg/mL	-	-	IV
Talimogene laherparepvec	Injection/suspension	2.44 mg sodium dihydrogen phosphate dihydrate, 15.4 mg disodium hydrogen phosphate dihydrate, 8.5 mg sodium chloride, 40 mg myoinositol, and 20 mg sorbitol	10^6^(PFU)/1 mL	10^6^(PFU)/1 mL (For initial dose), 10^8^(PFU)/1 mL (For subsequent dose)	-	SC/IL
Alipogene tiparvovec	Injection/solution	Potassium dihydrogen phosphate, potassium chloride, sodium chloride, disodium phosphate, and sucrose	3 × 10 12-genome copies/mL	3 × 10 12-genome copies/mL	-	IM
Lumasiran sodium	Solution	Sodium hydroxide and phosphoric acid	94.5 mg/0.5 mL	94.5 mg/0.5 mL	7.0	SC
Inclisiran sodium (284 mg)	Solution	Sodium hydroxide and con. phosphoric acid	189 mg/mL	284 mg/1.5 mL	-	SC
AMVUTTRA (Vutrisiran sodium)	Injection	0.7 mg sodium phosphate dibasic dihydrate, 0.2 mg sodium phosphate monobasic dihydrate, 3.2 mg sodium chloride	26.5 mg/0.5 mL	26.5 mg/0.5 mL	7.0	SC
Tozinameran	Suspension	0.09 mg 1,2-distearoyl-sn-glycero-3-phosphocholine, 0.05 mg 2[(polyethylene glycol)-2000]- N,N-ditetradecylacetamide, 0.43 mg (4-hydroxybutyl)azanediyl)-bis(hexane-6,1-diyl)bis(2-hexyldecanoate), 0.2 mg cholesterol), 0.01 mg potassium chloride, 0.01 mg monobasic potassium phosphate, 0.07 mg dibasic sodium phosphate dihydrate, 6 mg sucrose, and 0.36 mg sodium chloride	30 mcg of mRNA	0.5 mg/1 mL	6.9 to 7.9	IM
Elasomeran	Suspension	Lipids, cholesterol, 1.93 mg (SM-102, polyethylene glycol-2000, 1,2-distearoyl-sn-glycero-3-phosphocholine), 0.31 mg tromethamine, 1.18 mg tromethamine HCl, 0.043 mg acetic acid, 0.12 mg sodium acetate, dimyristoyl glycerol, and 43.5 mg sucrose	100 mcg of mRNA	0.2 mg/1 mL	7.0 to 8.0	IM

(IVI, intravitreal; SC, subcutaneous; IV, intravenous; IM, intramuscular; IL, intralesional; IOI, intraocular injection; IT, intrathecal.).

## Abbreviations

AAV: adeno-associated virus; API, active pharmaceutical ingredient; ASOs, antisense oligonucleotides; COVID-19, coronavirus disease-2019; DDSs, drug delivery systems; DSPC, 1,2-distearoyl-sn-glycero-3-phosphocholine; EMA, European Medicines Agency; EUA, emergency use authorization; FDA, United States Food and Drug Administration; IL, intralesional; IM, intramuscular; IOI, intraocular injection; IV, intravenous; IVI, intravitreal; IT, intrathecal; LNP, lipid nanoparticle; mRNA, messenger RNA; NA, nucleic acid; NPs, nanoparticles; PEG-2000­C-DMG, α-(3′-{[1,2-di(myristyloxy)propanoxy]-carbonylamino}propyl)-ω-methoxy, polyoxyethylene; PEG2000-DMG, 1,2-dimyristoyl-rac-glycero3-methoxypolyethylene glycol-2000; PS, phosphorothioate; ROS, reactive oxygen species; siRNA, short interfering RNA; SC, subcutaneous; SSO, splice switching oligonucleotide; Synthetic therapeutic oligonucleotides, STOs.
